# Addressing disability in the health system at CARITAS Takeo Eye Hospital

**Published:** 2013

**Authors:** Manfred Mörchen, Gail Ormsby, Te Serey Bonn, David Lewis

**Affiliations:** Consultant ophthalmologist and CBM medical advisor to CARITAS Takeo Eye Hospital, Cambodia.; PhD Candidate, Centre for Eye Research Australia, University of Melbourne.; Programme Director, CARITAS Takeo Eye Hospital, Cambodia.; Strategic Programmes Director, CBM Australia.

**Figure:**
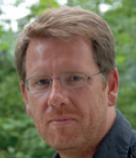
Manfred Mörchen

**Figure:**
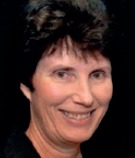
Gail Ormsby

**Figure:**
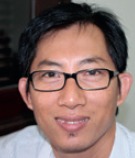
Te Serey Bonn

**Figure:**
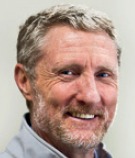
David Lewis

In recent years, CARITAS Takeo Eye Hospital (CTEH) in Cambodia has worked hard to be more inclusive of people with disabilities. While there have been some challenges along the way, the overall results of the new practices appear to be very positive.

The first change came in 2008, when the old, run-down eye hospital was replaced with a brand new facility. The major donor, CBM, encouraged the local partner CARITAS Cambodia to grasp the opportunity to design the new building so that it would meet high standards of accessibility. CBM emphasised that a ‘universal design’ approach, reducing the (physical) barriers for everybody, regardless of age and ability, could lead to a win–win situation for all patients, not only those with disabilities. Guidance from CBM (based on the document Promoting universal access to the built environment^1^) was invaluable for local architects, and the result was the construction of an eye hospital with significantly improved physical accessibility.

The second important change in strengthening practices related to people with disabilities, beyond just physical accessibility, was triggered by the Avoidable Blindness Initiative funded by the Australian Agency for International Development (AusAID). This programme emphasised wider issues including disability inclusion, gender, and child protection. Eye care projects had to report specifically against these issues, for example, physical accessibility for people with disabilities and the number of eye health services with documented referral pathways to disability services and disabled people's organisations.^2^ In collaboration with CBM Australia, a number of activities on different levels were implemented between 2009 and 2012:

- A ‘knowledge, attitude and practices’ (KAP) survey was conducted on people with and without self-reported impairment. This provided a ‘baseline’ or starting point from which to measure the hoped-for improvement.
- Training of local staff on inclusion – facilitated by a partnership between CBM Australia and the Nossal Institute for Global Health, University of Melbourne.
- In order to build and share knowledge, and to foster collaboration and partnership, workshops on disability inclusive practices were also conducted with local hospital staff, local provincial health authorities, community-based rehabilitation (CBR) organisations, partner eye care organisations, government officials and the National Program for Eye Health.
- A manual, called Disability-inclusive practices in eye health^3^, was written in collaboration with the CBM Australia-Nossal partnership and distributed to those involved in the work. A condensed, translated version was also distributed to all local health authorities in Takeo province.
- CTEH advocated for consideration of disability inclusive eye care practices into national eye health guidelines. As a result, Cambodia's National Programme for Eye Health – run by the ministry of health – made disability inclusion part of the national primary eye care curriculum from 2011.
- A key recommendation for improved disability inclusive practice in eye health relates to access to low vision services. CTEH has developed a low vision department, employing refractionist nurses trained to provide low vision services.
- Collaborations with both mainstream schools and specialist schools for blind children, relating to low vision and disability inclusion generally, have been strengthened.
- Improved collaboration with the local CBR organisation, Cambodian Development Mission for Disability. This has strengthened referral and support for people with disabilities and for other poor patients. This includes waiving user fees, transportation assistance, and distribution of food vouchers.
- The development of a computerised health information system with data collection on self-reported disabilities.

**Figure:**
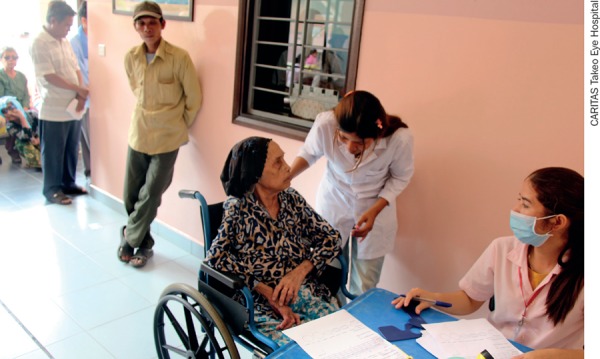
Better physical accessibility and greater awareness of disability by hospital staff have improved the inclusion of people with impairments. CAMBODIA

**BARRIERS FACED BY PEOPLE WITH DISABILITIES**

**Figure:**
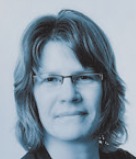
Diane Mulligan

Deputy Director, Advocacy and Alliances for Inclusive Development, CBM.

The specific identification and removal of barriers is the essence of accessibility. Barriers can be grouped into four categories:

1. **Physical or environmental barriers**. Access to buildings, schools, clinics, water pumps, transport, roads, paths, etc.
2. **Communication barriers**. Written and spoken information including media, flyers, internet, community meetings, etc.
3. **Policy barriers**. Including legislation that discriminates against people with disabilities, and/or an absence of legislation that might otherwise provide an enabling framework. Departmental and organisational policies can also be addressed here.
4. **Attitudinal barriers**. Including negative stereotyping of people with disabilities, social stigma and other forms of overt discrimination. It is not uncommon that disability is associated with cultural beliefs about sin, evil and witchcraft. People with disabilities often report that attitudes are the most disabling barriers of all.

The results from the KAP survey^4^ have been very useful, especially in highlighting the barriers which prevent all members of the population from accessing eye care. For example:

- Only 19percnt; of people with self-reported impairments (including those related to vision) reported being able to travel to the eye hospital on their own, whereas nearly twice as many people with no reported impairment (36%) claimed to be able to travel alone.
- Only 83percnt; of people with self-reported impairments said that they would look for treatment in case of an eye problem, compared with 95percnt; of people with no impairment.

The implementation of the new low vision department at CTEH has been especially successful. The refractionist nurses who were trained in low vision care received ongoing monitoring. They have been able to integrate the new service into outpatient department activities. In addition, rehabilitation of visually impaired patients in the hospital and through growing collaboration with mainstream and specialist schools is leading to improved outcomes for these patients.

The inclusion of a disability component in the new health information system raised several problems. These included the need for a simple definition of disability in this context (e.g. a definition of ‘hearing impairment’ in an environment where hearing tests are not available) and staff members’ concerns about the additional workload required. Asking patients to self-report any disabilities – for example by including the Washington Group's self-reporting questions^5^ in patient registration forms – is highly recommended, as it is both simple and efficient. CTEH is now able to provide evidence that a significant number of patients have other impairments in addition to visual impairment.

Overall, our efforts to strengthen disability-inclusive practices appear very worthwhile, but more research is certainly needed.
