# Supplementary material for: Addressing disability in the health system at CARITAS Takeo Eye Hospital
Source: Community Eye Health. 2013;26(81):8–9. (PMC3678309)
Supplement: Related texts

# Brief report: Disability: Recommendations for eye programmes

**Published:** 2013

In 2012, CBM's Medical Eye Care Advisory Working Group met in Hyderabad, India to discuss the inclusion of people with disabilities in eye care.

As a result of these discussions, the following recommendations were made:

- Involve local disabled people's organisations (DPOs) in planning (page 12).
- Appoint a member of staff as the coordinator for disability inclusion in all eye units (this may be a part time role).
- Identify barriers to access, both internal and external, noting which are easy and which are difficult to fix. Put in place an action plan to address these.
- Consider any additonal needs based on gender and age.
- Ensure that eye care services are comprehensive and include health promotion, disease prevention, curative medical and surgical services, and rehabilitation services.
- Ensure counselling, links and referrals to rehabilitation and education services are available to people who cannot be helped clinically. Ensure these services also refer patients to eye units.
- Staff with the heaviest loads – such as ophthalmologists – need to know that they can (in a caring manner) refer patients to other skilled staff in the unit.
- Employ people with disabilities to work in eye clinics.
- Ensure physical accessibility as well as large, colour-contrasting signage.
- Specifically plan for the provision of services for hearing impaired people.
- Include disability-inclusive practices in training curricula.
- Provide disability-inclusive training for eye unit staff and raise awareness with other stakeholders. For example, simply training staff to say: ‘I can refer you to XYZ, because there isn't anything more I can do for you' versus saying: ‘Nothing can be done for you’, can make an enormous difference to ‘quality of life’ or ‘whole of life’ outcomes and the mental health of patients with long-term visual impairment.

Colin Cook, Babar Qureshi, Harpreet Kapoor, Dominic Misquith, and David Lewis

**Improving access for women and girls with disabilities**

Nearly all eye health programmes strive to reach the most marginalised people. They also seek to be gender sensitive, ensuring equal access for all people. Women and girls with disabilities (including those with impaired vision) are some of the most marginalised people, as they face the triple discrimination of being female, having an impairment, and being among the poorest.

It is important that eye health programmes consider how they can support women with a disability. Here are some practical tips:

- Consult with women with disabilitito identify what is blocking their access to eye care, and to talk about how best to overcome these barriers
- Raise awareness among staff and collaborators about the impact of disability on women and girls and work together to address barriers
- Appoint a coordinator for disability inclusion, who understands gender sensitive practice (part-time or full-time)
- Collect and analyse data by gender, age, and disability, for example by using the Washington Group self-reporting questions.
- Employ women with a disability in your programme.
- Develop networks and two-way referral between your programme and primary health care, rehabilitation, education, and DPOs.
- Ensure that women and girls with disabilities who cannot be assisted through medical intervention are referred to other services, such as education, rehabilitation, livelihood, social inclusion and health services.

*With thanks to Joanne Webber, Chelsea Huggett, and CBM partners in India and Cambodia*.
